# Consumption of salmon fishmeal increases hepatic cholesterol content in obese C57BL/6 J mice

**DOI:** 10.1007/s00394-022-02930-y

**Published:** 2022-07-05

**Authors:** Marit Hjorth, Atanaska Doncheva, Frode Norheim, Stine Marie Ulven, Kirsten Bjørklund Holven, Thomas Sæther, Knut Tomas Dalen

**Affiliations:** 1 Department of Nutrition, Institute of Basic Medical Sciences, University of Oslo, Sognsvannsveien 9, Domus Medica, Blindern, P.O. Box 1046, 0317 Oslo, Norway; 2 Norwegian National Advisory Unit On Familial Hypercholesterolemia, Oslo University Hospital, Aker Sykehus, Postboks 4950, 0424 Oslo, Norway; 3 Department of Molecular Medicine, Institute of Basic Medical Sciences, University of Oslo, Sognsvannsveien 9, Domus Medica, 0372 Oslo, Norway; 4 The Norwegian Transgenic Center, Institute of Basic Medical Sciences, University of Oslo, Oslo, Norway

**Keywords:** Salmon, Fishmeal, Liver, Cholesterol, Glucose tolerance, RNA sequencing, Pcsk9, Salmon by-products, Fish protein

## Abstract

**Purpose:**

By-products from farmed fish contain large amounts of proteins and may be used for human consumption. The purpose of this study was to investigate cardiometabolic effects and metabolic tolerance in mice consuming fishmeal from salmon by-products, salmon filet or beef.

**Methods:**

Female C57BL/6J mice were fed chow, as a healthy reference group, or a high-fat diet for 10 weeks to induce obesity and glucose intolerance. Obese mice were subsequently given isocaloric diets containing 50% of the dietary protein from salmon fishmeal, salmon filet or beef for 10 weeks. Mice were subjected to metabolic phenotyping, which included measurements of body composition, energy metabolism in metabolic cages and glucose tolerance. Lipid content and markers of hepatic toxicity were determined in plasma and liver. Hepatic gene and protein expression was determined with RNA sequencing and immunoblotting.

**Results:**

Mice fed fishmeal, salmon filet or beef had similar food intake, energy consumption, body weight gain, adiposity, glucose tolerance and circulating levels of lipids and hepatic toxicity markers, such as p-ALT and p-AST. Fishmeal increased hepatic cholesterol levels by 35–36% as compared to salmon filet (*p* = 0.0001) and beef (*p* = 0.005). This was accompanied by repressed expression of genes involved in steroid and cholesterol metabolism and reduced levels of circulating Pcsk9.

**Conclusion:**

Salmon fishmeal was well tolerated, but increased hepatic cholesterol content. The high cholesterol content in fishmeal may be responsible for the effects on hepatic cholesterol metabolism. Before introducing fishmeal from salmon by-products as a dietary component, it may be advantageous to reduce the cholesterol content in fishmeal.

**Supplementary Information:**

The online version contains supplementary material available at 10.1007/s00394-022-02930-y.

## Introduction

A constantly increasing world population creates higher demands on food production and requires improved agricultural and aquacultural sustainability and efficiency [1]. This necessitates exploring potential use of food resources that are available but not used for human consumption. One such resource is by-products from fish [2, 3]. In Norway, one of the largest fishery nations in the world, around 27% of produced fish and seafood are discarded from human consumption and classified as by-products [4]. Farmed salmon by-products contain large amounts of proteins and other nutrients, and may serve as a significant food source of human nutrition [2, 3].

Fish has high content of protein and fatty acids (FAs), as well as several nutrients, such as vitamin D, iodine, selenium and taurine that are less abundant in many types of food typically consumed by humans. Compared to available food alternatives, fish products are particularly attractive for human consumption since intake of fish is associated with reduced risk of cardiovascular disease and may also protect against metabolic syndrome [5, 6]. These beneficial health effects are in part caused by a high content of marine n-3 polyunsaturated fatty acids (PUFA), such as docosahexaenoic acid (DHA) and eicosapentaenoic acid (EPA) with anti-inflammatory properties (reviewed in [7]). EPA and DHA can also affect membrane fluidity, thereby influencing lipid raft assembly and inflammatory signal transduction [7]. Furthermore, dietary FAs can modulate lipid metabolism by controlling transcription factors, such as peroxisome proliferator-activated receptors (PPARs) and sterol regulatory element binding proteins (SREBPs) [8, 9]. Marine n-3 PUFAs can suppress FA synthesis in liver via reduced SREBP-1c activity and increase FA oxidation via activation of PPARα and upregulation of FA-oxidizing enzymes. Due to these metabolic effects, intake of marine n-3 FAs can decrease hepatic lipid accumulation, VLDL secretion and plasma triglyceride levels.

While many studies support that consumption of marine FAs have beneficial metabolic effects in humans, the potential beneficial effects of fish protein consumption is less explored. Growing evidence is suggesting that the source of dietary proteins similarly can influence health and longevity in humans [10–13]. A high intake of animal protein, when compared against plant protein, has been associated with increased morbidity or mortality in several studies [10–13]. Furthermore, proteins from fish may have beneficial effects on metabolic health [14]. In a human intervention study, intake of dietary cod protein associated with increased insulin sensitivity [15]. In another intervention on overweight individuals, even a modest daily intake of a fish protein supplement (3–6 g) tended to improve glucose tolerance and LDL cholesterol [16]. In rodents, several studies have reported that salmon protein hydrolysates can improve glucose regulation, reduce body weight gain and modulate hepatic lipid metabolism [17–19].

We recently conducted a randomized, controlled trial on 74 human participants to investigate effects of 8 weeks supplementation with fishmeal from salmon by-products [20]. Daily supplementation with 7.5 g of fishmeal, corresponding to 5.2 g of salmon protein, did not influence cardiometabolic risk factors, such as glucose tolerance, serum lipids or blood pressure in humans. In the current study, the objective was to investigate metabolic effects of a high intake of fishmeal in obese mice. To investigate the effects of fishmeal feeding, compared to feeding with salmon filet or beef, obese C57BL/6 J mice were fed for 10 weeks with high-fat diets formulated with half of the protein content from these alternative protein sources.

## Materials and methods

### Ethical considerations and animal housing conditions

Experimental use of animals was approved by the Norwegian Animal Research Authority (Mattilsynet, approvals FOTS id: #10,902) and conformed to the ARRIVE guidelines and ethical guidelines in Directive 2010/63/EU of the European Parliament on the protection of animals used for scientific purposes. Mice were housed with a 12 h light/dark cycle (7 am to 7 pm), with 55% relative humidity at 22 °C, with free access to water and food. The presence of pathogens was monitored quarterly according to the FELASA guidelines (Federation of European Laboratory Animal Science Associations). Animals were specific pathogen-free according to FELASA recommendations (SPF status). A total of 40 mice were included in this study and no mice were excluded from the analyses.

### Preparation and nutritional composition analysis of freeze-dried ingredients

High-quality salmon fishmeal was produced from by-products of farmed Atlantic salmon, such as heads, viscera and spines (Mowi ASA, Bergen, Norway). Salmon filet from back loin (salmon filet) and entrecote of beef (beef) were freeze-dried in house (LyoQuest, Telestar, Spain) and ground in a regular kitchen mixer. Amino acid content (Supplemental Tables S1) and micronutrient content (Supplemental Tables S2) in fishmeal, salmon filet and beef were measured by Eurofins Food & Feed Testing Norway AS.

FA composition in fishmeal, salmon filet and beef was measured by VITAS analytical services (Forskningsparken, Norway); freeze-dried samples were weighed and transferred to soda lime tubes. Internal standard, methyl tricosanoate and 3 N Methanolic HCl were added and FAs methylated for 2 h at 80 °C prior to analysis. 100 µl prepared sample was transferred to GC vial, diluted with hexane and injected on an Agilent 7890A Gas Chromatograph System (Agilent Technologies). FAs were separated on a Supelcowax (30 m  × 250 µm × 0.2 µm) column.

### Mouse diet intervention

**Fig. 1:**
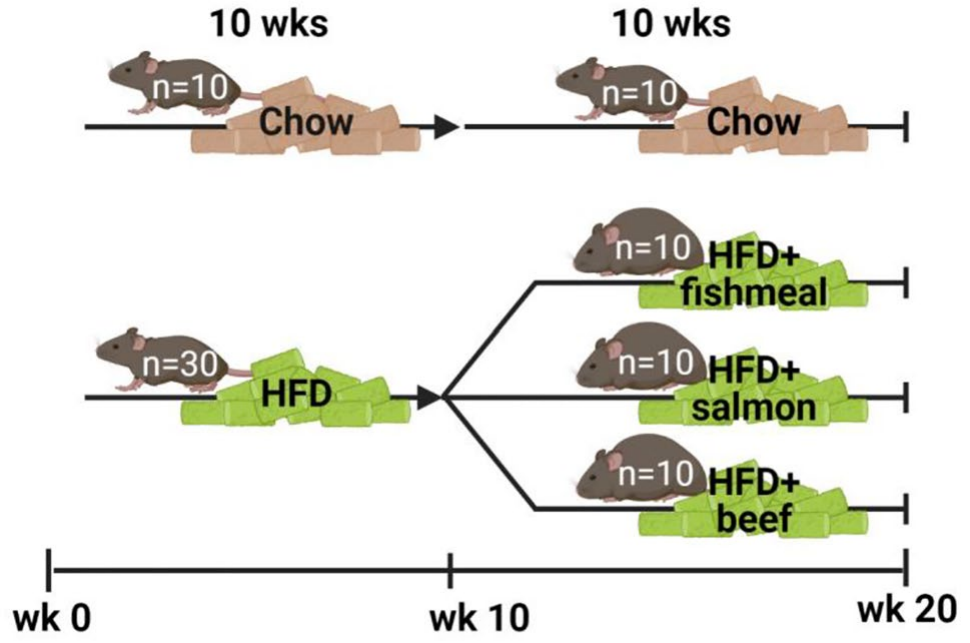
Experimental design. Female C57BL/6 J mice were fed chow (*n* = 10) or a high-fat diet (HFD, *n* = 30) from 8 weeks of age (wk 0) until 18 weeks of age (wk 10). HFD-fed mice were subsequently randomized to receive HFDs containing protein from three alternative sources; salmon fishmeal extracted from salmon by-products (fishmeal), salmon filet from back loin (salmon filet) or entrecote of beef (beef) until 28 weeks of age (wk 20). The alternative HFD diets contained 50% of protein from casein and 50% protein from fishmeal, salmon or beef. Chow-fed mice continued on the chow diet. Indirect calorimetry was performed in week 17, glucose tolerance testing (GTT) was performed in week 19 and mice were euthanized in week 20. Body weights and body composition were determined weekly and at multiple time points, respectively. The figure was created with BioRender.com

Female C57BL6/J mice (8 weeks, Janvier Labs) were fed a chow diet with 11 E% from fat, 62 E% from carbohydrates and 26 E% from protein (Special Diets Service, UK, #RM3) or a high-fat diet (HFD) with 60 E% from fat, 20 E% from carbohydrates and 20 E% from protein (Research diets, cat#D12492) for 10 weeks. From 18 to 28 weeks of age, chow-fed mice continued on the chow diet, while the HFD-fed mice were split into three groups and given alternative HFDs that differed in their sources of protein (Table 1, Fig. 1); fishmeal extracted from salmon by-products (Mowi ASA; fishmeal); salmon filet from back loin (salmon filet) or entrecote of beef (beef). The alternative diets were generated by modifying the HFD into a basis diet lacking 10 E% protein from casein (Research diets, cat#D19020401), which were supplemented with 10 E% protein from freeze-dried fishmeal, salmon filet or beef (Table 1). Soybean oil was added to adjust for differences in fat content in the freeze-dried protein sources (Table 1) to generate isocaloric diets with the same amounts of carbohydrates, protein and fat (Table 2). The three diets were mixed in a regular kitchen mixer, sterilized by autoclaving, formed into pellets by hand and stored at  – 20 °C until use. The drinking water was supplemented with multivitamin drops (Vitamin A, D3, E, K3 and B-vitamin complex) for rodents.

**Table 1:** Nutrient composition of freeze-dried fishmeal, salmon filet and beef

	Fishmeal powder	Salmon filet powder	Beef powder
Energy, kcal/100 g	398	597	572
Protein, g/100 g	70	58	68
Fat, g/100 g	13	41	33
Carbohydrate g/100 g	< 0.1	< 0.1	< 0.1
Sodium, g/100 g	0.65	0.16	0.19
Cholesterol, g/100 g	0.49	0.14	0.19

**Table 2:** Composition of diets supplemented with fishmeal, salmon filet or beef

Content (g/100 g diet)	Fishmeal diet	Salmon filet diet	Beef diet
Test ingredient*	18.8	22.7	19.4
*- Protein content in test ingredient*	*13.1*	*13.1*	*13.1*
*- Fat content in test ingredient*	*2.5*	*9.3*	*6.5*
Casein	13.1	13.1	13.1
L-Cystine	0.4	0.4	0.4
Maltodextrin 10	16.4	16.4	16.4
Sucrose	9.0	9.0	9.0
Soybean oil**	10.1	3.3	6.1
Lard	32.2	32.2	32.2
Water	0.0	2.9	3.5
Cholesterol	0.115	0.054	0.059
Energy, kcal/100 g	610	610	610
Protein, E%	17	17	17
Carbohydrate, E%	17	17	17
Fat, E%	66	66	66
Saturated fat, g^§^	14.6	14.4	17.0
Monounsaturated fat, g^§^	17.8	20.2	18.5
Polyunsaturated fat, g^§^	10.4	8.4	7.3
Omega-3 fatty acids, g^§^	1.25	1.25	0.75
Omega-6 fatty acids, g^§^	8.44	5.15	6.41

Content of test ingredients (fishmeal or freeze-dried salmon filet and beef), and other ingredients contributing with protein, fat and carbohydrates (g/100 g). Total dietary content of fat, protein, carbohydrates, cholesterol and energy

^*^Fishmeal from salmon by-products, salmon filet or beef. Ingredients were freeze-dried, and contributed with 50% of the dietary protein

^**^Added to adjust for variation in fat content

^§^Approximate fatty acid distribution in the final diets was estimated from fatty acid content in soybean oil and lard (nutritiondata.self.com) and measured fatty acid levels in freeze-dried fishmeal, salmon or beef

At 28 weeks of age, mice were sedated by isoflurane inhalation and blood was collected by cardiac puncture with an EDTA-coated syringe. This was followed by cervical dislocation and rapid collection of tissues. Tissues were snap frozen in liquid nitrogen. Plasma was obtained by separating out blood cells with gentle centrifugation.

### Metabolic phenotyping, GTT and body composition measurements

Body composition measurements were performed at multiple time points with a Minispec LF90II TD-NMR machine (Bruker, Billerica, MA, USA). Body weight was measured weekly. Food intake was measured by weighing food consumed in each cage at 16 individual time points.

At 25 weeks of age, mice were placed in a metabolic cage system consisting of 20 individual cages (Phenomaster, TSE Systems, Germany) for 3 days. For indirect calorimetric measurement of gas exchange (oxygen consumption and carbon dioxide production), we used the following settings: gas flow rate at 0.42 l/minute, measurement time for 10 s, and gas exchange measurement in 20 min intervals. Physical activity was measured as movement in the XY-plane. Body weight and composition were measured prior to metabolic phenotyping.

At 27 weeks of age, glucose tolerance testing (GTT) was performed. Mice were fasted for 5 h, starting at 08:00 am, and then injected (i.p) with 2 g glucose (20% glucose in saline) per kg lean mass. Blood samples were collected from the tip of the tail before glucose injection, and 15, 30, 45, 60, 90 and 120 min after glucose injection. Glucose concentrations were measured in whole blood with a FreeStyle Precision Neo glucometer (Abbott, IL, US). Insulin concentrations were measured in 5 µl plasma with ELISA (Crystal Chem, cat# 90,080).

### Analyses of cholesterol, triglycerides, bile acids and liver enzymes

Preparation of liver lysates: Liver tissue was homogenized in 5% NP40 in PBS by vigorous shaking with glass beads with Precellys 24 (Bertin Technologies, Rockville MD US) and then sonicated on high power mode for 2 cycles, 30 s on and 30 s off with a Bioruptor plus (Diagenode, Belgium). Protein concentrations in liver lysates were measured with a Pierce BCA assay (Thermo Fisher Scientific, Waltham, MA US).

Triglycerides and total cholesterol in liver lysates and plasma were measured with colorimetric reagent sets (Pointe Scientific, Canton, MI, US, Cat# T7532 and Cat# C7510). HDL and LDL/VLDL cholesterol in plasma were measured with a colorimetric kit (Abcam, cat# ab65390). Plasma concentrations of alanine transaminase (ALT) and aspartate transaminase (AST) were measured with colorimetric MedTest reagent sets (Pointe Scientific, Canton, MI, US, Cat# A7561/Cat# A7526).

Bile acids were measured in plasma, liver and faeces with a total bile acids kit for mouse (Crystal Chem, cat# 80,470). Faeces was collected from individually housed mice and air-dried for 4 days. Liver and faeces samples were homogenized in 75% ethanol in water by vigorous shaking with glass beads (Precellys 24, Bertin Technologies, Montigny-le-Bretonneux, France). They were then incubated for 2 h at 50 °C in glass tubes and centrifuged (10 min, 6000 g, 4 °C).

### RNA isolation, reverse transcription and quantitative PCR

Liver tissue was homogenized in RA1 lysis buffer by vigorous shaking with glass beads (Precellys 24, Bertin Technologies, Montigny-le-Bretonneux, France). RNA was subsequently isolated using a NucleoSpin RNA isolation kit (Macherey–Nagel, Allentown, PA, US), with a modified pre-preparation method prior to loading on the RNA-binding columns [21]. RNA purity and concentration was measured on a NanoDrop ND-100 Spectrophotometer (Thermo Fisher Scientific, Waltham, MA US).

*Reverse transcription (RT) of RNA.* Total RNA was reversely transcribed with random hexamers using a High Capacity cDNA Reverse Transcription Kit (Thermo Fisher Scientific, Waltham, MA US, Cat. #4,368,814) on an ep Gradient S Eppendorf Mastercycler (Eppendorf AG, Hamburg, Germany) with the following settings: 25 °C for 10 min, 37 °C for 120 min, 85 °C for 5 min and 4 °C on hold.

*qPCR with fluorescent intercalating dye.* Gene-specific regions were amplified from cDNA with assay primers (200 nmol/L each) and Bio-Rad SsoAdvanced™ Universal SYBR® Green Supermix (10 µL reaction, 95 °C for 3 min, followed by 40 cycles of 95 °C for 10 s and 60 °C for 20 s) on a CFX96 Touch instrument (Bio-Rad). Primers used were designed with Primer-BLAST (Supplemental Table S3). Gene expression was calculated using the 2^−∆∆Ct^ method. Tata-binding protein (*Tbp*) mRNA was verified to be stably expressed among groups and treatments, and was used as a reference gene.

### RNA sequencing and data analysis

RNA integrity was determined using Bioanalyzer RNA 6000 Nano (Agilent Technologies), according to the manufacturer’s instruction. The RNA samples had RIN values between 8.1 and 8.7. Samples were sequenced at the Norwegian Sequencing Centre (Oslo University Hospital, Ullevål, Oslo). Total RNA samples were subjected to Strand-Specific TruSeqTM mRNA-seq library preparation, and 50 bp paired-end reads were sequenced on an Illumina Novaseq 600 instrument.) To remove/trim low-quality reads and adapter sequences we used BBDuk (BBMap v34.56 [22]. Reads were mapped to the *Mus musculus* reference genome (ENSEMBL release 101, GRCh38.101) using HiSat2 v.1.2.1 [23]. Read counting was done with FeatureCounts v1.4.6-p1 [24]. The average read count was 40 million/sample. Raw sequencing data and normalized counts are available in the GEO repository (GSE188546).

### Immunoblotting

Liver tissue was lysed in RIPA buffer containing cOmplete EDTA-free Protease Inhibitor Cocktail (Cat. #11,836,170,001, Roche, Basel, Switzerland) and phosphatase inhibitors (Cat. #P0044, Sigma-Aldrich, St. Louis, MO, US). Samples were homogenized using glass beads and a Precellys 24 tissue homogenizer (Bertin Instruments, Montigny-le-Bretonneux, France) and sonicated on a Bioruptor® Plus device (Diagenode, Liege, Belgium). Lysates were diluted in Laemmli buffer, proteins separated on Criterion™ TGX™ 4–20% gels (Bio-Rad, Hercules, CA, US), and transferred to nitrocellulose membranes using the Trans-Blot Turbo transfer system and RTA transfer kit (Bio-Rad, Hercules, CA, US). Membranes were stained with Ponceau S to confirm similar total protein loaded in each lane. Membranes were blocked in Tris-buffered saline containing 0.1% Tween-20 (TBS-T) and 5% BSA and incubated over night with primary antibodies in TBS-T containing 2.5% BSA. After washing, membranes were incubated with appropriate HRP-conjugated secondary antibodies. Primary antibodies used were as follows: Goat anti-Ldlr (#AF2255, R&D Systems), goat anti-Pcsk9 (#AF3985, R&D Systems, Minneapolis, MN, US), rabbit anti-Hmgcr (#PA5-37,367, Thermo Fisher Scientific, Waltham, MA US), and rabbit anti-Gapdh (#sc-25778, Santa-Cruz Biotechnology, Dallas, TX, US). Secondary antibodies used were as follows: Goat anti-rabbit IgG (#111–035-144), and bovine anti-goat IgG (#805–035-180; Jackson ImmunoResearch, West Grove, PA, US). Chemiluminescence detection was done on the ChemiDoc™ Touch Imaging System (BioRad, Hercules, CA, US). Band intensities were quantified relative to Gapdh signals using ImageJ v1.52 software (NIH, Bethesda, MD, US).

### Data analysis

For analyses of RNA sequencing counts we used R version 4.0.3. Differential expression analyses were done using the Deseq2 pipeline on background filtered read counts [25]. False discovery rate (fdr) < 0.05 was considered statistically significant. PCA plots were generated after variance-stabilizing transformation in Deseq2. Gene ontology analyses were done in the clusterProfiler package with KEGG pathway annotations [26]. For other analyses, we used Graphpad Prism software (version 8). Methods used for statistical testing are listed in the figure legends.

## Results

### Nutritional composition of diets supplemented with fishmeal, salmon or beef

To study the effects of fishmeal, salmon or beef intake in obese and glucose intolerant animals, 8-week-old female C57BL/6 J mice were first fed a high-fat diet (HFD, 60 E% from fat) for 10 weeks (Fig. 1). As expected, the HFD feeding led to increased body weights and fat mass (9.7 g fat mass, compared to 0.92 g in the chow group), as well as enhanced plasma blood glucose before and 1 h after intraperitoneal administration of glucose (Supplemental Fig. 1). The obese mice were then randomized to receive a HFD containing 50% of total protein from freeze-dried fishmeal, salmon filet or beef for 10 weeks (Fig. 1). To generate alternative HFDs diets that were isocaloric with equal amounts of carbohydrates, fat and protein, we first measured the content of macronutrients in each protein source. Freeze-dried fishmeal contained 69.7% protein, while salmon and beef contained 57.7% and 67.8% protein, respectively. Salmon filet contained more fat (40.8%) compared to fishmeal (13.2%) and beef (33.4%), while carbohydrate content was marginable (Table 1). Soybean oil was added to diets to adjust for differences in fat content in the alternative protein sources (Table 2). The remaining fat in the HFDs was mainly from lard, while carbohydrates were mainly from sucrose and maltodextrin.

**Fig. 2:**
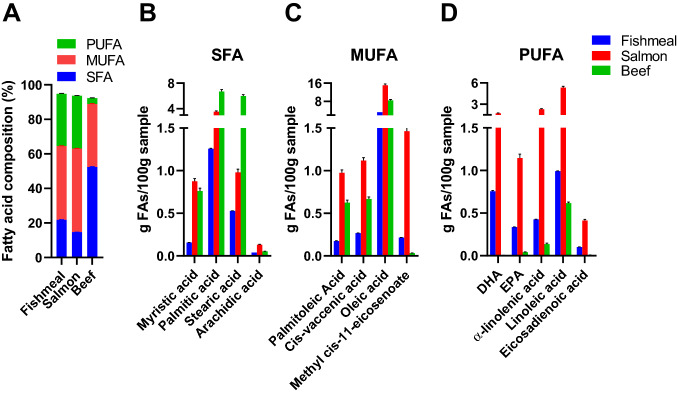
Fatty acid content in fishmeal, salmon filet and beef. Gas chromatography determination of individual fatty acid (FA) content in freeze-dried fishmeal, salmon filet and beef used to supplement the different HFDs. In total, 11 saturated FAs (SFA), 6 monounsaturated FAs (MUFA), 10 polyunsaturated FAs (PUFA) and 5–8% uncharacterized FAs (not shown) were detected. Ingredients were measured in in triplicate. Analytical variation was ~ 2% for abundant FAs and maximum 7% for rare FAs. **a** Fatty acid (FA) composition of SFA, MUFA and PUFA expressed as % of total FA content. **b–d** Concentrations of the most abundant SFAs, MUFAs and PUFAs expressed as grams of FAs per 100 g of freeze-dried protein extract (*n* = 3)

We further performed detailed analyses of macro- and micronutrients in freeze-dried fishmeal, salmon filet and beef (Table 1, Supplementary Table S1-2, Fig. 2). As expected, a higher proportion of the FAs in beef were saturated FAs (SFA; 52%), compared to 15% SFA in salmon filet and 22% SFA in fishmeal. The amount of monounsaturated FAs (MUFA) was more similar, while in both fishmeal and salmon filet, approximately 30% of the fat was PUFAs (Fig. 2a). Salmon filet contained a higher amount of marine PUFAs (> C20), including DHA and EPA, compared to fishmeal and beef (Fig. 2c–d). Because freeze-dried salmon filet contained more fat than fishmeal and beef (see Table 1), salmon filet contained higher amounts of both MUFAs and PUFAs as compared to fishmeal and beef (Fig. 2c–d). FAs from freeze-dried material and the added soybean oil constituted ~ 28% of total fat in the final diets, while the rest of the fat was from lard. Although levels of SFAs, MUFAs and PUFAs were different in the three freeze-dried ingredients, the levels of these FAs in the final diets were more comparable (Table 2). Of other lipids, fishmeal contained higher levels of cholesterol (493 mg/100 g) compared to salmon filet (144 mg/100 g) and beef (190 mg/100 g; Table 1). This resulted in > 2-fold higher content of total cholesterol in the fishmeal diet (Table 2).

Because different amino acids have different effects on liver metabolism, we compared the amino acid content of freeze-dried fishmeal, salmon filet and beef (Supplementary Table S1). Essential amino acid content was quite similar, with the exception of higher levels of histidine in beef. Fishmeal contained more glycine (5.1 g/100 g) compared to salmon filet (2.6 g/100 g) and beef (3.3 g/100 g). Fishmeal also contained more proline (3.2 g/100 g) than salmon filet and beef (2.0 and 2.7 g/100 g, respectively), as well as hydroxyproline (0.57 g/100 g). The latter being below limit of detection for salmon filet and beef. A higher content of glycine, proline and hydroxyproline is most likely originating from collagen in, e.g. connective tissue, a major part of salmon by-products.

Lastly, we measured the content of vitamins and minerals in salmon fishmeal (Supplementary Table S2) and compared against corresponding reference values for salmon filet and beef (Norwegian Food Composition Database [27]). Fishmeal contained high levels of vitamin B_2_ (5.1 mg/100 g), vitamin B_12_ (61 μg/100 g) and several minerals, particularly calcium (2500 mg/100 g), zinc (140 mg/100 g), copper (8.6 mg/100 g) and iodine (0.17 mg/100 g). The high content of copper and zinc in fishmeal leads to intake levels of these minerals above the recommended upper limit for human consumption (calculated as mg/kg body weight) in mice fed fishmeal [28]. In sum, several amino acids as well as other nutrients, such as cholesterol and several minerals, were enriched in fishmeal and could potentially influence metabolism.

### High-fat diets supplemented with fishmeal, salmon or beef have similar effects on adiposity

**Fig. 3:**
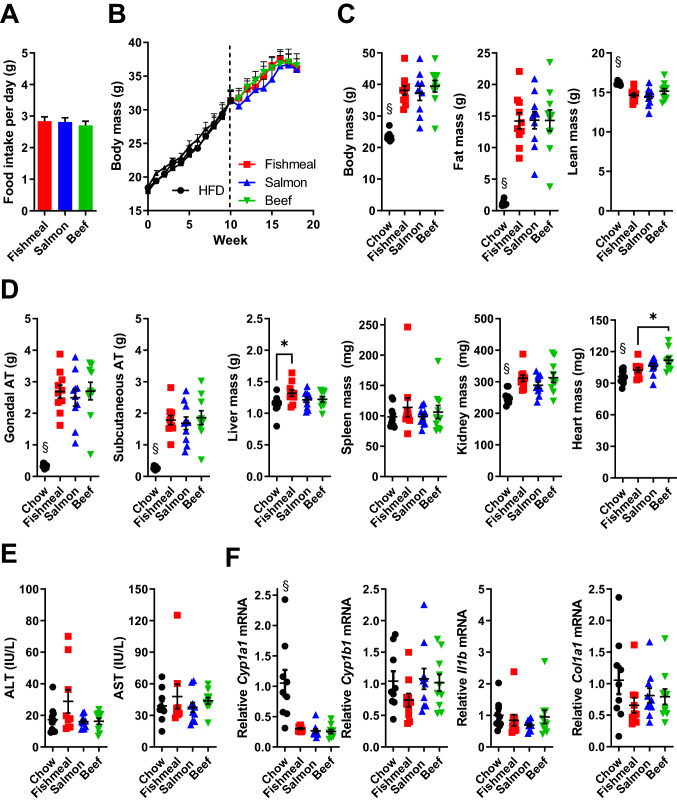
Adiposity and organ weights in mice receiving a HFD supplemented with fishmeal, salmon or beef. Determination of total body weight, organ weights and hepatic toxicity in female C57BL/6 J mice fed chow or alternative HFDs containing fishmeal, salmon filet or beef (*N* = 10 per group). Animals were terminated in week 20 (28 weeks of age) and organs weighted at dissection. RNA isolated from liver was analysed with RT-qPCR. **a** Food intake for HFD groups, measured cage wise at 16 different time points expressed as grams per mouse per day. **b** Total body weights in HFD groups from week 0 until week 20. **c** Body weights, lean mass and fat mass in individual mice at 28 weeks of age (body composition was determined with minispec LF90II TD-NMR). **d** Organ weights for gonadal adipose tissue (AT), intraperitoneal subcutaneous AT, liver, spleen, kidney and heart at 28 weeks of age. **e** Plasma levels of alanine transaminase (ALT) and aspartate transaminase (AST). One blood sample from the fishmeal group was excluded due to hemolysis, and one sample was excluded from the AST graph due to technical error. **f** Hepatic gene expression of xenobiotic and drug metabolizing enzymes (*Cyp1a1*, *Cyp1b1*), inflammation (*Il1b*) and fibrosis (*Col1a1*). Statistical testing was done with one-way ANOVA and Tukey’s or Dunnett’s test for multiple comparisons. ^§^*p* < 0.05 between chow and at least two other groups. *p < 0.05 between indicated groups

To determine if all diets were consumed and well tolerated by the mice, we measured food intake by weighing food at multiple time points throughout this study. In average, food consumption was slightly below 3 g/mouse/day, with no difference between the dietary groups (Fig. 3a). To investigate the effects of fishmeal, salmon filet or beef consumption on body weight and adiposity, we measured body weight weekly (Fig. 3b). After the first 10 weeks on the regular HFD, the average body weight was approximately 31 g, and this increased to approximately 38 g after another 10 weeks on a HFD containing fishmeal, salmon filet or beef (Fig. 3b). There were no differences between the HFD groups in body weight gain or body weight at the end of the study, or between lean mass and fat mass measured by NMR (Fig. 3c). Feeding with fishmeal, salmon filet or beef did not influence weights of tissues and organs, such as gonadal and subcutaneous (inguinal) adipose depots, liver, spleen or kidney (Fig. 3d). Mice fed fishmeal tended to have slightly heavier livers, but this difference was only statistically significant when comparing to chow-fed mice (Fig. 3d, *p* = 0.12 when comparing fishmeal and salmon filet groups). As expected, several of the parameters measured above were different between the three HFD groups and the metabolically normal chow-fed group. Chow-fed mice had a lower total body weight, higher lean mass and lower fat mass (Fig. 3c), and lower adipose tissue mass, kidney and heart organ weights (Fig. 3d).

To further determine if all diets were tolerated, we measured plasma levels of markers associated with liver damage. There was no difference in plasma ALT and AST (Fig. 3e). Two mice in the fishmeal group displayed higher levels of AST or ALT, but there were no statistically significant differences between the groups. We also measured hepatic expression of genes involved in detoxification/drug metabolism (*Cyp1a1*, *Cyp1b1*), inflammation (*Il1b*) and hepatic fibrosis (*Col1a1*). There were essentially no differences in expression of these markers (Fig. 3f), except for a reduction in *Cyp1a1* mRNA expression in all HFD groups.

### Fishmeal, salmon or beef consumption did not influence metabolic rates or glucose tolerance

**Fig. 4:**
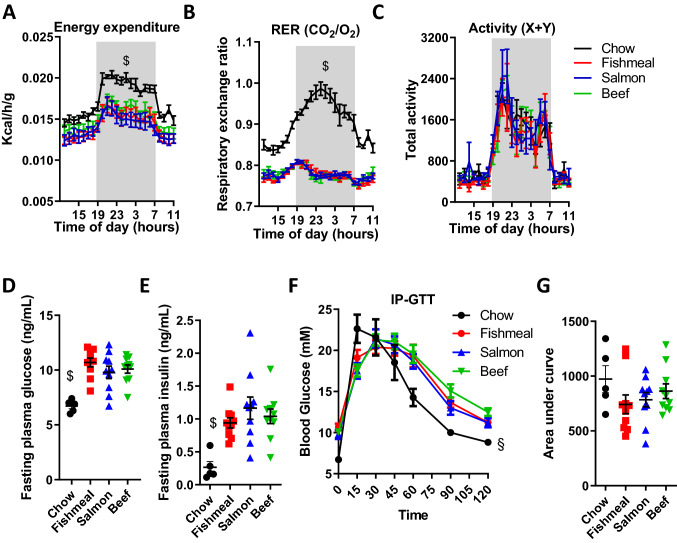
Energy expenditure and glucose tolerance in mice receiving a HFD supplemented with fishmeal, salmon or beef. Mice fed a chow or a high-fat diet supplemented with fishmeal, salmon or beef were subjected to metabolic phenotyping (week 17) and glucose tolerance testing (week 19). **a** Energy expenditure per gram bodyweight. **b** Respiratory exchange ratio. **c** Total activity levels in the XY-plane. **d** Plasma glucose, and **e** plasma insulin concentrations after 6 h fasting. **f**–**g** Glucose tolerance testing displayed as plasma glucose concentration for up to 120 min after intraperitoneal glucose injection and area under curve. Statistical testing was done with one-way ANOVA (**d**, **e**, **g**) or two-way ANOVA (**a**–**c**, **f**) and Tukey or Dunnett’s test for multiple comparisons. ^$^*p* < 0.05 between chow and at least two other groups. ^§^*p* < 0.05 between chow and the other groups at 0, 60, 90 and 120 min

To assess the metabolic effects of dietary fishmeal, salmon filet or beef, all dietary groups were placed in metabolic chambers after 7 weeks on the supplemented diets (week 17). As expected, chow-fed mice had a higher energy expenditure per gram body weight, and a higher respiratory exchange ratio compared to HFD-fed mice (Fig. 4a-b). Consumption of fishmeal, salmon filet or beef did not lead to alterations in metabolic rates, and we did not observe any differences in physical activity levels (Fig. 4c).

We further investigated glucose metabolism by performing glucose tolerance testing after 9 weeks on the supplemented diets (Fig. 4d–g). Chow-fed mice had significantly lower fasting blood glucose and plasma insulin. After an i.p. injection of glucose (2 g/kg lean mass), chow-fed mice got a large increase in blood glucose, but the glucose clearance was faster compared to mice in the three HFD supplemented groups (Fig. 4f). There was no difference in glucose tolerance between mice receiving HFD supplemented with fishmeal, salmon filet or beef (Fig. 4f-g).

### High-fat feeding leads to substantial transcriptomic changes in liver

**Fig. 5:**
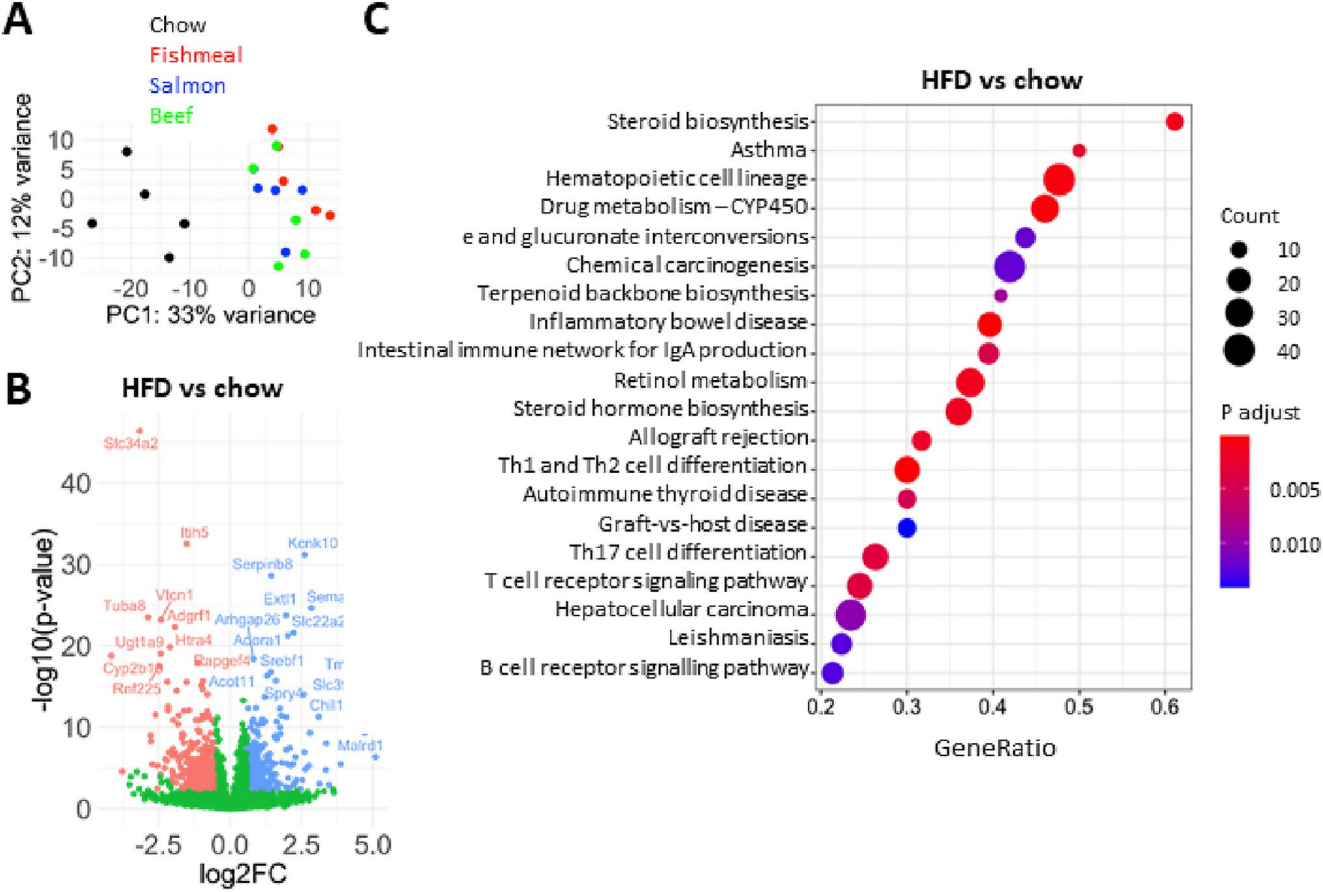
High-fat feeding leads to large transcriptomic changes in liver. Total RNA from mice fed chow or HFDs containing fishmeal, salmon filet or beef (*N* = 5 per group) were subjected to RNA sequencing. **a** Principal component analysis of the global gene expression after variance-stabilizing transformation. The chow-fed mice are clearly segregated from the three HFD groups. **b** Differential gene expression analysis of HFD-fed mice versus chow-fed mice. Data from the three HFD groups (fishmeal, salmon filet and beef) were pooled. Genes upregulated in the HFD groups are indicated in blue (FC > 1.5, fdr < 0.05), and downregulated genes are indicated in red (FC < -1.5, fdr < 0.05). **c** Gene set enrichment analysis of differential gene expression between HFD-fed mice and chow-fed mice. Top 20 enriched KEGG pathways are shown. Colours indicate adjusted *p*-values and circle sizes indicate gene counts for the given pathways

Although the three protein sources seemed well tolerated and did not seem to affect weight gain, hepatic function and glucose clearance, they could potentially alter metabolic pathways. Thus, we measured global gene expression in liver of all four dietary groups with RNA sequencing of five representative individuals from each diet group. As expected, principal component analysis revealed a distinct gene expression pattern in chow-fed mice, while there was no separation between the HFD groups supplemented with fishmeal, salmon filet or beef (Fig. 5a). We first compared the liver transcriptome of HFD-fed mice (pooled data from fishmeal, salmon filet and beef groups) to chow-fed mice. Differential gene expression analysis was performed on 15,713 protein coding genes (Supplementary Table 4a). In total, 1818 genes were differentially expressed (fdr < 0.05), of which 341 genes were > 1.5-fold upregulated and 373 genes were > 1.5-fold downregulated (Fig. 5b).

To identify the molecular pathways that were altered with HFD feeding, we performed a gene set enrichment analysis (Fig. 5c). Several of the most enriched KEGG pathway were related to steroid or terpenoid biosynthesis, including steroid biosynthesis, steroid hormone biosynthesis and terpenoid backbone biosynthesis. Many of the differentially expressed genes in these pathways are involved in cholesterol synthesis (*Msmo1*, *Cyp51*, *Nsdhl*, *Fdft1*, *Lss*, *Hsd17b7*, *Lbr*, *Idi1*, *Hmgcs1*, *Acat2*). Furthermore, many genes belonged to the CYP family, whereof some were related to arachidonic acid or retinol metabolism. The differentially expressed genes in these pathways were downregulated in HFD-fed mice, suggesting a reduced cholesterol synthesis, and potentially reduced metabolism of steroids, arachidonic acid or retinol.

Other regulated KEGG pathways were related to inflammation and immune regulation, including the pathways for asthma, hematopoietic cell lineage, inflammatory bowel disease and Th1 and Th2 cell differentiation (Fig. 5c). Of the specific genes were markers of T-cells or other immune cells (including *Cd4*, *Cd3*, *Cd28*, *Cd2*, *Cd22*, *Cd5*, *Cd6* and *Cd79*), or interleukins and interleukin receptors (including *il1b*, *il12rb2*, *il18rap*, *il2rg*, *il21r*, *il7r* and others). Interestingly, these genes were significantly downregulated in all groups of HFD-fed mice.

To ensure that these gene expression changes were not primarily due to either fishmeal, salmon filet or beef consumption, we also performed differential gene expression analysis of each individual dietary group compared to the chow group (fishmeal vs. chow, salmon filet vs. chow and beef vs. chow; Supplemental Table 4b–d). We further performed gene set enrichment analysis of KEGG pathways, which revealed large overlaps between the different comparisons (Supplemental Fig. 2). Again, pathways related to steroid metabolism and the immune system were among the most enriched pathways for fishmeal, salmon filet or beef compared to chow-fed mice. These data indicate that the primary effect on hepatic gene expression was the HFD in itself, and not the alternative sources of protein.

### Consumption of fishmeal leads to reduced hepatic expression of genes involved in cholesterol and steroid biosynthesis

**Fig. 6:**
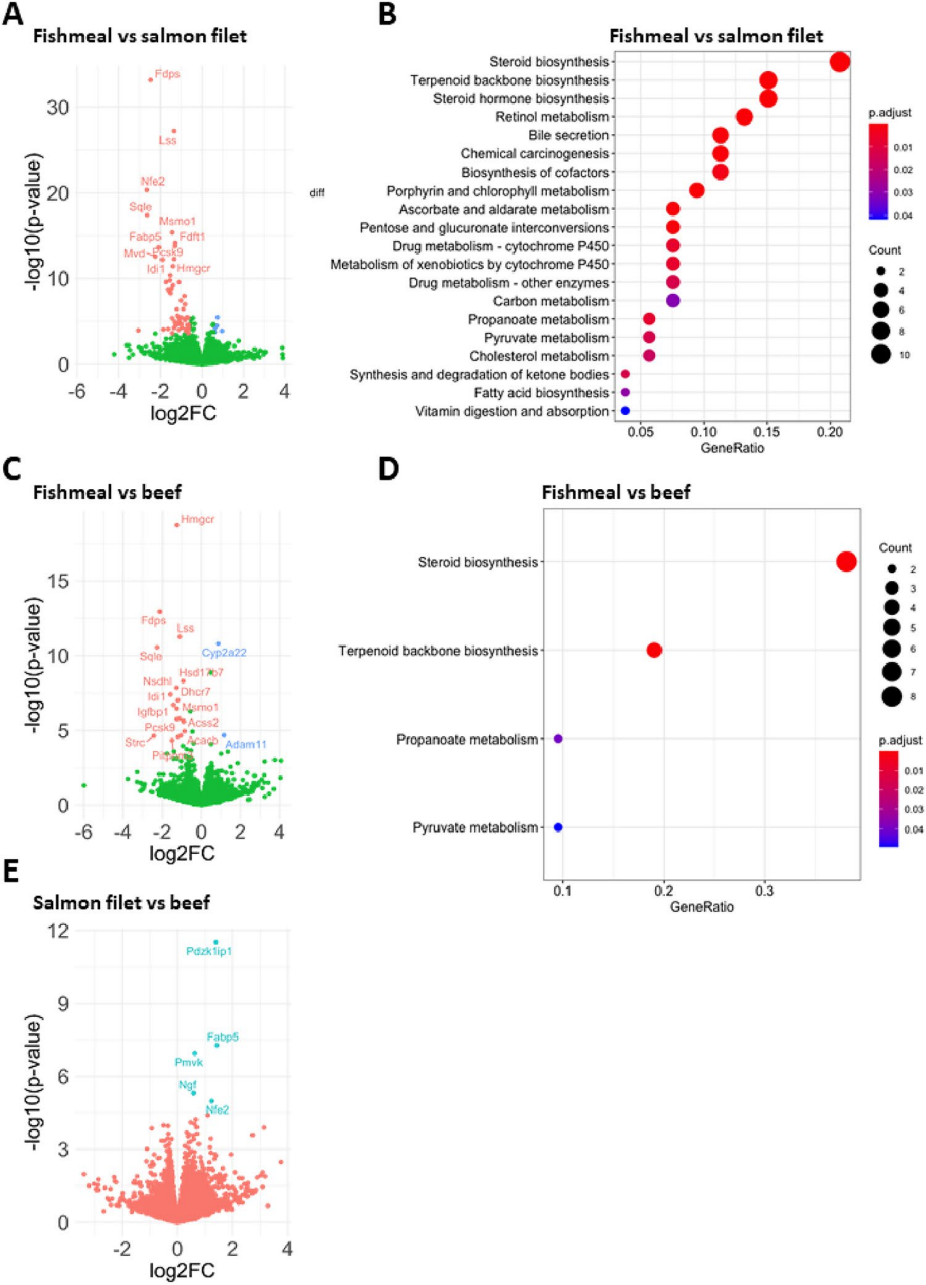
Fishmeal consumption leads to a downregulation of genes related to steroid metabolism in liver. Differential hepatic gene expression analysis between mice fed HFD containing fishmeal, salmon filet or beef. **a** Differential hepatic gene expression analysis of mice fed fishmeal versus salmon filet. Genes upregulated in fishmeal fed mice are indicated in blue (FC > 1.5, fdr < 0.05) and downregulated genes are indicated in red (FC < -1.5, fdr < 0.05). **b** Overrepresentation analysis of genes downregulated in mice fed fishmeal compared to salmon filet. The most enriched KEGG pathways are shown; colours indicate adjusted *p*-values and circle sizes indicate gene counts for the given pathways. **c** Differential hepatic gene expression analysis of mice fed fishmeal versus beef (colour coded as described in A). **d** Overrepresentation analysis of genes downregulated in mice fed fishmeal compared to beef (colour coded as described in B). **e** Differential gene expression analysis of mice fed salmon filet versus beef. Genes that were upregulated in the salmon group are indicated in green (FC > 1.5, fdr < 0.05). *N* = 5 per group

To further investigate the hepatic effects of fishmeal, we performed differential gene analysis between the HFD groups. First we compared gene expression in livers of mice fed fishmeal and salmon filet. In total, 76 mRNAs were differentially expressed (fdr < 0.05), whereof 56 were downregulated and 6 were upregulated (fold change > 1.5) in the fishmeal group (Fig. 6a, Supplementary Table S5a). Several of the downregulated genes were involved in cholesterol or sterol biosynthesis synthesis, including the rate-limiting enzyme HMG-CoA reductase (*Hmgcr*). To identify molecular pathways that were altered, we performed a KEGG pathway overrepresentation analysis of genes that were differentially expressed with an fdr < 0.1. Again, the most downregulated pathways were related to cholesterol and steroid biosynthesis, drug metabolism as well as metabolism of other components, such as retinol, pentose/glucuronate, pyruvate or FAs (Fig. 6b).

Next, we compared gene expression in livers of mice fed fishmeal and beef (Fig. 6c). We identified 27 genes that were differentially expressed (fdr < 0.05, Supplementary Table S5b). Only two of these genes were > 1.5-fold increased (*Cyp2a22*, *Adam11*), while 20 genes were > 1.5-fold downregulated. KEGG pathways enriched among the differentially expressed genes were steroid biosynthesis, terpenoid backbone biosynthesis, propionate metabolism and pyruvate metabolism (Fig. 6d). Overall, consumption of fishmeal led to downregulation of many of the same genes and pathways when compared to either salmon filet or beef.

Lastly, we compared gene expression in livers of mice fed salmon filet and beef (Fig. 6e). Despite larger differences in FA composition between beef and salmon filet (see Table 2), only 5 protein coding genes were differentially expressed (fdr < 0.05, Supplementary Table S5c). *Pdzk1ip1*, *Fabp5*, *Nfe2*, *Pmvk* and *Ngf* were all upregulated in mice fed salmon filet compared to beef (FC > 1.5). This did not allow for gene set enrichment analyses. To conclude from all comparisons, steroid synthesis-related pathways in particular seemed repressed in the livers of mice receiving fishmeal.

### Fishmeal consumption increased the accumulation of cholesterol in liver

**Fig. 7:**
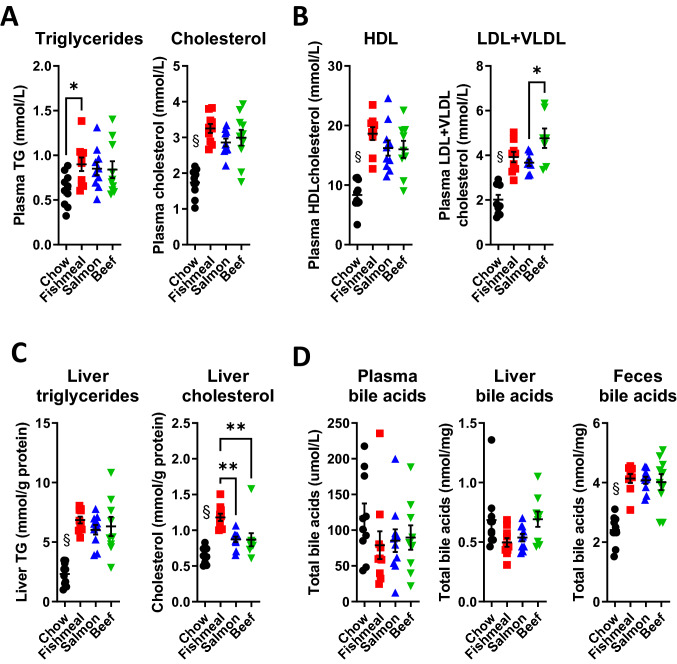
Fishmeal feeding leads to increased hepatic cholesterol levels. Content of triglycerides, cholesterol and bile acids in mice fed fishmeal, salmon filet or beef. **a** Plasma triglycerides and cholesterol levels. **b** HDL cholesterol and LDL/VLDL cholesterol levels. **c** Hepatic triglycerides and cholesterol levels. **d** Concentrations of total bile acids in plasma, liver and feces. Statistical testing was done with one-way ANOVA and Tukey’s or Dunnett’s test for multiple comparisons. §*p* < 0.05 between chow and at least two other groups. *p < 0.05, **p < 0.01 between indicated groups

Several studies have shown that both protein from fish as well as marine FAs can modify circulating lipids and metabolism of lipids in liver [14]. Our discovery of a likely repression of the steroid and cholesterol biosynthesis pathways in mice receiving fishmeal, prompted us to follow up with detailed analysis of circulating and hepatic lipids. As expected, feeding with all HFDs increased plasma concentrations of triglycerides, total cholesterol, HDL cholesterol as well as LDL and VLDL cholesterol (Fig. 7a–b). Similarly, high-fat feeding also increased hepatic levels of triglycerides and total cholesterol, as compared to chow-fed mice (Fig. 7c). Levels were similar between the HFD groups, but interestingly, mice fed fishmeal had higher levels of hepatic cholesterol (Fig. 7c) as compared to mice fed salmon filet (35% increase, *p* = 0.002) or beef (36% increase, *p* = 0.002). Dietary fishmeal also tended to increase the concentration of plasma cholesterol (Fig. 7a) as compared to salmon filet (unadjusted *p* = 0.029, not significant after adjustment for multiple testing).

To investigate if hepatic alterations in cholesterol levels were reflected in altered bile acid concentrations, we measured total bile acids in plasma, liver and faeces. Plasma bile acid concentrations were highly variable, and there were no significant differences in bile acids between the groups (Fig. 7d). No differences were observed in bile acid concentration in livers among the groups; however, feeding with HFD increased the concentration of bile acids in faeces as compared to feeding with chow.

### High cholesterol content in fishmeal results in hepatic repression of cholesterol metabolism

**Fig. 8:**
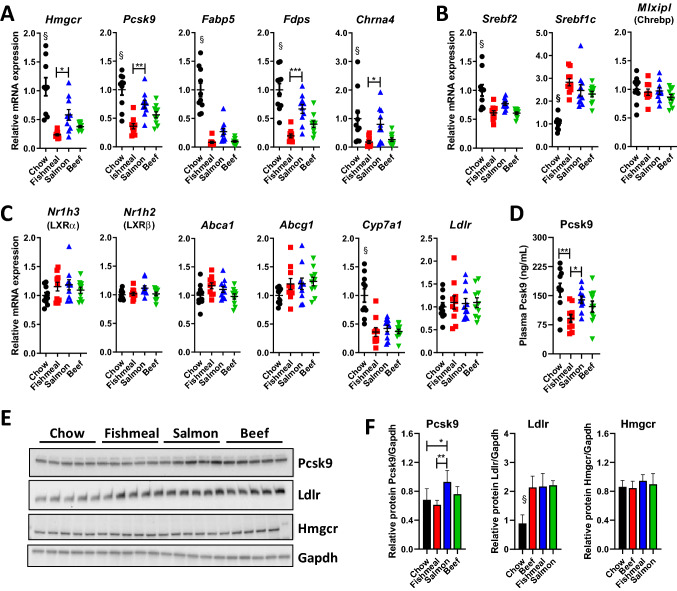
Fishmeal feeding leads to circulating PCSK9. Expression of hepatic genes important for steroid hormone synthesis and cholesterol metabolism in mice fed fishmeal, salmon filet or beef (*n* = 10). **a** Validation RNA sequencing analysis with RT-qPCR analysis of selected genes identified as low expressed in fishmeal compared to salmon or beef (*Hmgcr*, *Pcsk9*, *Fabp5*, *Fdsp*, *Chrna4*). **b** Expression of mRNAs encoding genes regulating cholesterol synthesis (*Srebf2*) and lipogenesis (*Srebf1c* and *Mlxipl*/Chrebp). **c** Expression of cholesterol metabolite sensors (*Nr1h3*/LXRα and *Nr1h2*/LXRβ), cholesterol efflux transporters (*Abca1* and *Abcg1*) and the rate-limiting enzyme in bile acid synthesis (*Cyp7a1*). **d** Plasma PCSK9 protein levels. **e**–**f** Immunoblot analysis and relative quantification of hepatic Pcsk9, Ldlr, Hmgcr and Gapdh protein levels (*n* = 5). Statistical testing was done with one-way ANOVA and Tukey test for multiple comparisons. $*p* < 0.05 between chow and at least two other groups. *p < 0.05, *p < 0.01 between indicated groups

To further investigate why mice receiving fishmeal had elevated hepatic cholesterol, we analysed expression of genes important for hepatic cholesterol uptake, synthesis and excretion. RT-qPCR analysis with all individuals included pr. group (*n* = 10) also functioned to verify our transcriptomic analyses that identified several genes in the cholesterol and sterol biosynthesis pathways as repressed in fishmeal compared to salmon filet or beef (see Fig. 6a). Both *Hmgcr* and *Fdps*, which are involved in cholesterol and sterol biosynthesis, were downregulated in fishmeal compared to salmon filet-fed mice (Fig. 8a). *Pcsk9*, an important regulator of LDL receptor degradation, was also downregulated in mice fed fishmeal compared to salmon. Interestingly, while HFD seemed to suppress expression of hepatic genes involved in cholesterol uptake and synthesis, the effect was stronger when the diet was substituted with fishmeal compared to salmon filet.

Next, we measured expression of transcription factors known to stimulate cholesterol synthesis (*Srebf2*) and lipogenesis (*Srebf1c* and *Mlxipl*/Chrebp), and transcription factors that are activated when cholesterol metabolites accumulates in liver (*Nr1h3*/LXRα and *Nr1h2*/LXRβ) to stimulate hepatic removal of cholesterol by activating the cholesterol efflux transporters (*Abca1* and *Abcg1*) and the rate-limiting enzyme in bile acid synthesis (*Cyp7a1*). Several of these genes were differently expressed between chow and the HFD groups, but no differences in expression were observed between the fishmeal, salmon filet and beef groups (Fig. 8b–c).

Based on our gene expression analysis, we centred our attention on hepatic uptake of LDL, since several genes in this process were expressed at lower levels in the fishmeal group compared to salmon filet. Plasma levels of the Pcsk9 protein, as well as hepatic expression of Pcsk9 protein, were lower in fishmeal compared to the chow or salmon filet groups (Fig. 8d–f). Hepatic protein expression of Ldlr was similarly elevated in all HFD groups compared to chow, whereas protein expression of Hmgcr was similar in all groups. Although these analyses are insufficient to give a clear explanation for the observed higher content of hepatic cholesterol with fishmeal, it suggests that fishmeal represses Pcsk9 expression, which in turn may affect hepatic Ldlr recycling and/or hepatic cholesterol uptake.

## Discussion

In this study, we used mice to investigate metabolic effects of dietary intake of protein from salmon fishmeal and two protein sources that are traditionally used in the human diet. Custom-made HFDs, with half of the dietary protein from fishmeal, salmon filet or beef, were given obese and glucose intolerant female mice. These mice were compared to reference mice given a standard rodent chow diet. All HFDs seemed well tolerated, based on equal consumption of food, animal growth curves, energy substrate oxidation, glucose tolerance, plasma lipids and markers of hepatic toxicity. Still, mice fed fishmeal had alterations in the hepatic transcriptome and increased liver cholesterol, which was accompanied by a tendency for increased liver weight and plasma cholesterol. Thus, we concentrated our molecular analysis on dissecting changes in hepatic cholesterol metabolism and pathways involved.

The high-fat diets were isocaloric and adjusted to contain equal amounts of carbohydrates, protein and fat. Still, detailed analysis of the protein supplements revealed differences in amino acid composition, certain vitamins and minerals, FA saturation and cholesterol content. The source of dietary proteins may influence metabolism by differences in amino acid content and bioactive peptides [3, 14, 29]. Fishmeal contained high levels of glycine, proline and hydroxyproline compared to salmon filet and beef, probably due to a high collagen content. None of these are essential amino acids. Collagen peptides are reported to have antioxidant properties as well as angiotensin I-converting enzyme inhibitory properties [3], while supplementation with glycine may improve glucose tolerance in humans and rodents [30]. Our transcriptomic analysis revealed no clear differences in hepatic expression of genes in antioxidant signalling pathways, and glucose clearance was similar between the three diets.

With respect to vitamins and minerals, fishmeal contained high levels of vitamin B_12_, copper, zinc, calcium and iodine. These nutrients are not suspected to alter hepatic cholesterol levels, but particularly for copper, the high levels resulted in intake above recommended levels for human consumption (normalized to mg/kg body weight). The Nordic Nutrition Recommendations from 2012 recommend an upper intake level of 5 mg copper pr. day [28], which for an adult human will be reached with a daily intake of 58 g of fishmeal. We did not observe any sign of copper toxicity with this diet for 10 weeks, but the high content of zinc may have functioned as a chelating agent and prevented copper uptake [31]. Nevertheless, the high content of several nutrients in fishmeal must be considered if salmon fishmeal is to be used as an ingredient in food for human consumption.

With respect to the FA composition, freeze-dried fishmeal and salmon filet contained low levels of SFA compared to beef. Fishmeal also contained higher levels of certain marine PUFAs (EPA and DHA) compared to beef, but levels were low compared to salmon filet. Studies in rodents have shown that intake of omega FAs can increase, while intake of saturated FAs can decrease insulin sensitivity [32]. We observed no differences in glucose uptake in our study, as assessed by GTT, suggesting that all diets affected insulin signalling similarly. PUFAs may further signal to influence lipid metabolism and inflammation [7–9], and prevent hepatic cholesterol accumulation [33]. Interestingly, the observed hepatic cholesterol levels in our study are in line with effects mediated by differences in PUFA content between freeze-dried fishmeal and salmon filet, but not when compared to beef, suggesting that levels of PUFA alone cannot explain increased cholesterol accumulation with fishmeal. Importantly, all three diets contained high levels of SFA from lard, which may mask a potential metabolic response caused by differences in PUFAs present at low levels.

In addition to differences in FAs, cholesterol content was higher in freeze-dried fishmeal compared to salmon filet and beef, resulting in 0.11% versus ~ 0.05% cholesterol in the final diets, respectively. To determine if the increased dietary intake of cholesterol with fishmeal was responsible for elevated hepatic cholesterol levels, we analysed pathways important for cholesterol metabolism. Hepatic cholesterol levels are balanced by several pathways; uptake of cholesterol-rich lipoproteins, de novo cholesterol synthesis, cholesterol excretion into the bile and conversion of cholesterol into bile acids. Mice will regulate these pathways to accommodate changes in dietary cholesterol and are efficient in clearing excess cholesterol when needed. Mice fed a diet containing 20-fold higher dietary cholesterol (2%) than our fishmeal diet (0.11%) for ten weeks increased hepatic cholesterol content ~ threefold [34]. In these mice, excessive cholesterol resulted in reduced expression of the rate-limiting enzyme in de novo cholesterol synthesis (*Hmgcr*), increased expression of the rate-limiting enzyme in conversion of cholesterol into bile acids (*Cyp7a*) and increased faecal bile acid secretion [34]. In our study, expression of *Hmgcr* was reduced in mice given fishmeal compared to mice given salmon filet, but we observed no differences in activation of LXRs and induction of cholesterol efflux (*Abca1* and *Abcg1*) or bile acid synthesis (*Cyp7a*) compared to chow diet. This suggests that the higher cholesterol content in the HFDs compared to chow mainly repress de novo cholesterol synthesis. Still, there seemed to be some differences also with respect to cholesterol uptake between the diets. Cholesterol uptake is mediated by three different receptors [35, 36]; LDL receptor (*Ldlr*; uptake of LDL and chylomicron remnants), the LDL receptor-related protein (*Lrp1*; uptake of chylomicron remnants) and Scavenger receptor class B type 1 (*Scarb1*, uptake of HDL). In the current study, hepatic expression and circulating levels of Pcsk9 were reduced with fishmeal compared to mice given salmon filet. Reduced levels of Pcsk9 are expected to favour recycling and prevent lysosomal degradation of Ldlr leading to increased Ldlr-mediated LDL uptake at the plasma membrane [37]. Despite the altered Pcsk9 levels, hepatic mRNA and protein expression of Ldlr as well as plasma levels of HDL and LDL + VLDL were similar between fishmeal, salmon filet and beef. This points to alterations in chylomicron remnants as the casual driver for elevated hepatic cholesterol with fishmeal; the higher cholesterol content in fishmeal may increase cholesterol-rich chylomicron remnants taken up by liver, resulting in hepatic cholesterol accumulation and repression of de novo cholesterol synthesis.

Several studies have suggested potential health benefits of fish proteins, but the available data are unclear. It has been reported that low-dose cod protein supplementation have potential favourable health effects on glucose metabolism [15, 16] and LDL cholesterol [16], although supplementation with salmon, cod or herring protein did not influence markers of glucose tolerance when compared to milk protein in another study [38]. We recently tested the fishmeal used in this study in a randomized trial on pre-diabetic human participants [20]. Dietary supplementation with a modest dose of fishmeal (7.5 g/d) for 8 weeks was well tolerated but did not influence cardiometabolic health markers or glucose clearance compared to placebo [20]. Since human trials examining health effects of lean and fatty fish protein supplements given at modest doses have variable results, a main objective of this rodent study was to investigate health effects with high intake of fishmeal. We did not observe differences in glucose clearance in obese mice with high intake (half of the protein) from fishmeal, salmon filet or beef. Our results in mice contrast several previous studies in rodents that have indicated positive metabolic health effects of diets containing lean or fatty fish, fish protein or fish protein hydrolysates [17–19, 39–42]. Rats fed a high-fat diet supplemented with cod had improved insulin sensitivity compared to rats fed casein or soy [39]. Mice fed a Western diet supplemented with lean seafood (scallop and fish filet) had reduced fat mass and improved glucose metabolism compared to lean meat supplementation, possibly due to lower feed intake and higher activity levels [42]. Rats fed protein hydrolysates from bonito, herring, mackerel or salmon had reduced expression of inflammatory cytokines in adipose tissue compared to casein [17]. Moreover, mice fed salmon protein hydrolysate gained less weight and had increased insulin sensitivity compared to casein-fed mice [17]. Collectively, these rodent studies suggest that not only marine FAs but also fish proteins mediate the beneficial health effects of fish. It is not clear why we are unable to replicate the above findings, but our study was designed differently. Our diet intervention started in obese mice, enabling us to circumvent effects on development of adiposity, with differences in body weight as a mediator of effectiveness on glucose clearance. Furthermore, food consists of many different macro- and micronutrients that may potentially signal independently of the tested ingredients, in our case proteins. Our carefully designed isocaloric diets, resulting in equal energy consumption of energy substrates (carbohydrates, protein and fat) and may have limited several confounding factors not necessarily accounted for in previous rodent studies.

In conclusion, our study reveals no sign of toxicity of fishmeal in mice, when constituting 50% of total dietary protein, but results in a significant rise in hepatic cholesterol. The biological mechanism behind the elevated hepatic cholesterol should be dissected further before fishmeal is used for human consumption. Lastly, industrial production of fishmeal with lower levels of cholesterol and certain minerals may be necessary before salmon by-products can be endorsed as a dietary component suitable for human consumption at higher doses.

## Supplementary Information

Below is the link to the electronic supplementary material.

Supplementary file1 (DOCX 1342 KB)

Supplementary file2 (XLSX 9784 KB)

Supplementary file3 (XLSX 6090 KB)

## Data Availability

Gene expression data described in this study are available in the NCBI GEO repository (https://www.ncbi.nlm.nih.gov/geo/query/acc.cgi?acc=GSE188546, accession number GSE188546). Remaining data that support the findings of this study are available from the corresponding author upon reasonable request.

## Abbreviations

Hmgcr: 3-Hydroxy-3-methylglutaryl *coenzyme A* reductase
ALT: Alanine transaminase
AST: Aspartate transaminase
DHA: Docosahexaenoic acid
EPA: Eicosapentaenoic acid
FA: Fatty acids
GTT: Glucose tolerance testing
HFD: High-fat diet
Ldlr: Low-density lipoprotein receptor
MUFA: Monounsaturated fatty acids
PPARs: Peroxisome proliferator-activated receptors
PUFA: Polyunsaturated fatty acids
Pcsk9: Proprotein convertase subtilisin/kexin 9
SFA: Saturated fatty acids
SREBPs: Sterol regulatory element binding proteins
Tbp: Tata-binding protein
