# Supplementary material for: Consumption of salmon fishmeal increases hepatic cholesterol content in obese C57BL/6 J mice
Source: Eur J Nutr. 2022 Jul 5;61(8):4027–43. doi: 10.1007/s00394-022-02930-y (PMC9596588; doi:10.1007/s00394-022-02930-y)
Supplement: Supplementary file 1 — Supplementary file1 (DOCX 1342 KB) [file 394_2022_2930_MOESM1_ESM.docx]

**Supplemental Fig. S1**

**

**

Supplementary Fig. 1. High fat diet feeding leads to obesity and reduced glucose tolerance.

Mice were fed a chow diet or a high fat diet for 10 weeks.

**a)** Body weight was measured weekly.

**b-c)**. Lean mass and fat mass (g) was measured with NMR.

**d-e)** Blood glucose after 5 hours fasting, and 1 hour after injection (i.p) of glucose (2 g/kg lean mass).

Statistical testing was done with a repeated measures ANOVA (A) or a students T-test. *p<0.05, ***p<0.001, ****p<0.0001.

**Supplemental Fig. S2**


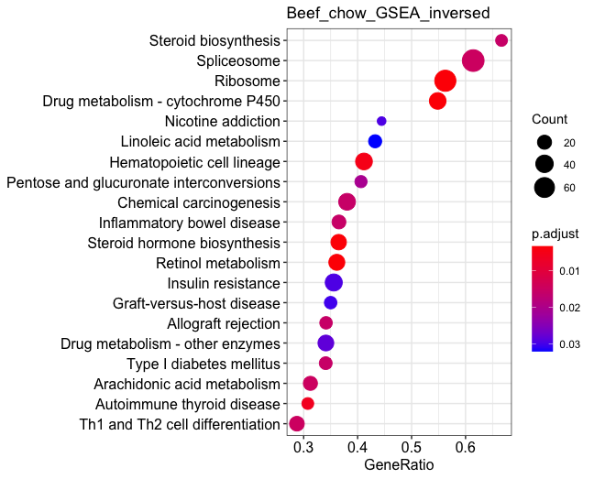

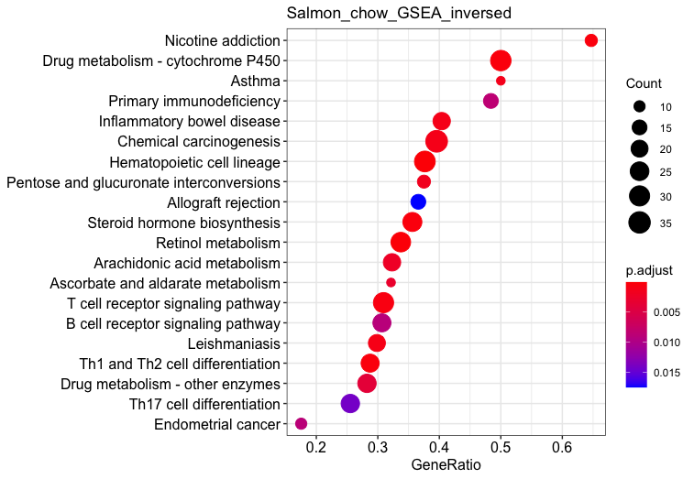

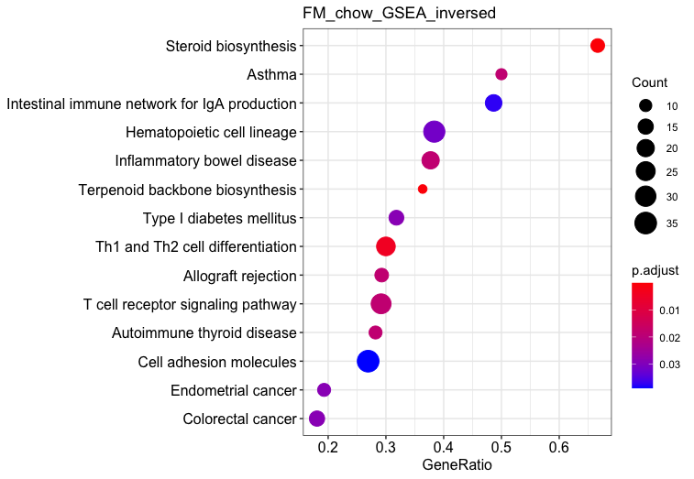


**A**

**B**

**C**

Fishmeal vs. chow

Salmon vs. chow

Beef vs. chow

**Supplementary Fig. 2. Gene set enrichment analysis against chow diet**

Gene set enrichment analysis (KEGG pathways) of differentially expressed genes in liver of mice fed a HFD supplemented with either fishmeal, salmon filet or beef, versus mice fed a chow diet.

**a)** Fishmeal versus chow.

**b)** Salmon filet versus chow.

**c)** Beef versus chow.

Supplementary Table S1. Amino acid content in freeze dried fishmeal, salmon filet and beef (g/100 g).

|  | **Fishmeal** | **Salmon filet** | **Beef** |
| --- | --- | --- | --- |
| ***Essential AA*** |  |  |  |
| Leucine | 4.6 | 4.3 | 4.9 |
| Isoleucine | 2.5 | 2.5 | 2.7 |
| Histidine | 1.5 | 1.5 | 2.4 |
| Lysine | 4.6 | 5.1 | 5.5 |
| Methionine | 1.8 | 1.7 | 1.6 |
| Phenylalanine (Total) | 2.5 | 2.4 | 2.5 |
| Threonine | 2.9 | 2.6 | 2.7 |
| Tryptophan | 0.87 | 0.77 | 0.85 |
| Valine (Total) | 3.2 | 2.9 | 3.0 |
| ***Non-essential AA*** |  |  |  |
| Alanine | 3.9 | 3.3 | 3.7 |
| Arginine (Total) | 4.0 | 3.3 | 4.0 |
| Aspartic acid | 5.8 | 5.5 | 5.7 |
| Cysteine + Cystine | 0.75 | 0.63 | 0.68 |
| Glutamic acid (Total) | 7.6 | 7.5 | 9.3 |
| Glycine | 5.1 | 2.6 | 3.3 |
| Proline (Total) | 3.2 | 2.0 | 2.7 |
| Serine (Total) | 2.9 | 2.1 | 2.4 |
| Tyrosine (Total) | 2.0 | 1.9 | 2.0 |
| Hydroxyproline | 0.57 | <0.05* | <0.05* |
| Ornithine | 0.067 | <0.05* | <0.05* |

*Below limit of detection.

Supplementary Table S2. Micronutrient content of salmon fishmeal

| **Nutrient per 100 g** | **Fishmeal** | **Salmon filet #** | **Beef #** |
| --- | --- | --- | --- |
| **Vitamins (per 100 g)** |  |  |  |
| Vit B_1_, thiamine (mg) | 0.15 | *0.32* | *0.12* |
| Vit B_2_, riboflavin (mg) | **5.1** | *0.30* | *0.33* |
| Vit B_3_, Niacin (mg) | 14 | *20* | *13* |
| Vit B_5_, pantothenic acid (mg) | 6.2 | *-* | *-* |
| Vit B_6_, pyridoxine hydrochloride (mg) | 0.34 | *1.4* | *1.3* |
| Vit B_8_, Biotin (µg) | 219 | *-* | *-* |
| Vit B_9_, Folate (mg) | 0.60 | *0.019* | *0.012* |
| Vit B_12_, cyanocobalamin (µg) | **61** | *9.5* | *3.3* |
| Vit D_3_, cholecalciferol (µg) | 4.8 | *27* | *0.61* |
| Vit E, alpha-Tocopherol (mg) | 8.1 | *3.8* | *1.5* |
| **Minerals (per 100 g)** |  |  |  |
| Calcium (mg) | **2500** | *19* | *12* |
| Copper(mg) | **8.6** | *0.11* | *0.18* |
| Iodine (mg) | **0.17** | *0.01* | *0.01* |
| Iron (mg) | 11 | *0.81* | *6.4* |
| Magnesium (mg) | 140 | *70* | *58* |
| Mercury (mg) | 0.011 | *-* | *-* |
| Potassium (mg) | 960 | *1219* | *1039* |
| Sodium (mg) | 650 | *124* | *133* |
| Selenium (mg) | 0.23 | *0.081* | *0.024* |
| Tin (mg)* | BD | *-* | *-* |
| Zinc (mg) | **140** | *1.4* | *14* |
| Values for fishmeal are analyzed by Eurofins.  * BD: Below limit of detection (<0.021 mg).  # Values for Salmon and Beef are based on reference values and adjusted for water content for the given foods in the ‘Norwegian Food Composition Database 2020’, Norwegian Food Safety Authority, [www.matvaretabellen.no](http://www.matvaretabellen.no). Values for salmon are based ‘*Salmon, farmed, raw*’ (containing 63 % water). Values for beef are based ‘Beef, *rib-eye steak, raw*’ (containing 67 % water). Nutrients with high content in fishmeal compared to Salmon filet or beef are shown in bold. | | | |

Supplementary Table S3. Primers used for RT-qPCR

| Gene symbol | Accession # | Forward primer | Reverse primer | Product size | Intron length |
| --- | --- | --- | --- | --- | --- |
| Abca1 | NM_013454.3 | ACCGAGGAAGAAGCTCGATG | GGTCGGGAGATGAGATGTGG | 103 | 11328 |
| Abcg1 | NM_009593.2 | AAGGTCTCCAATCTCGTGCC | CCCTGATGCCACTTCCATGA | 96 | 2131 |
| Chrna4 | NM_015730.5 | ATCTGGAGGCCTGACATCGT | TGCACACGCCCATCATAGAA | 98 | 4347 |
| Col1a1 | NM_007742.4 | CTGACGCATGGCCAAGAAGAC | CCTCGGGTTTCCACGTCTCA | 88 | 1465 |
| Cyp1a1 | NM_001136059.2 | CGTTATGACCATGATGACCAAGA | TCCCCAAACTCATTGCTCAGAT | 62 | 0 |
| Cyp1b1 | NM_009994.1 | CCAGATCCCGCTGCTCTACA | TGGACTGTCTGCACTAAGGCTG | 77 | 729 |
| Cyp7a1 | NM_007824.2 | TCCACTTCATCACAAACTCCCT | CTGTGTCCAAATGCCTTCGC | 109 | 694 |
| Fabp5 | NM_001272097.1 | GGAAGGAGAGCACGATAACAAG | GAAGCCCTCATTGCACCTTCT | 108 | 496 |
| Fdps | NM_001253751.1 | ATGCTATTGCCCGGCTCAAG | TCCTGGAAGGCTTGTACCAC | 91 | 1228 |
| Hmgcr | NM_001360165.1 | GCCTTGTGATTGGAGTTGGC | ACACTGACATGCAGCCGAAG | 78 | 2804 |
| Il1b | XM_006498795.3 | GCTGAAAGCTCTCCACCTCA | TGTCGTTGCTTGGTTCTCCT | 89 | 1149 |
| Ldlr | NM_010700.3 | GACTGCAAGGACATGAGCGA | TGTCCAAGCTGATGCACTCC | 103 | 1860 |
| Mlxipl | NM_021455.4 | TGCAGCCCAGCCTAGATGAC | AGCTGGGGGACTCTATGTAGTT | 102 | 4495 |
| Nr1h3 | NM_001177730.1 | GACTTCAGTTACAACCGGGAAGA | ATTCATGGCTCTGGAGAACTCAAA | 90 | 5129 |
| Nr1h2 | NM_001285517.1 | GAAGGCGTCCACCATTGAG | AAGTCGTCCTTGCTGTAGGT | 108 | 473 |
| Pcsk9 | NM_153565.2 | TGTCACAGAGTGGGACCTCA | CTCGGCCAGGGTAAGTGTG | 92 | 1179 |
| Srebf2 | NM_033218.1 | TGACTCTCGGGGACATCGAC | CACCTCCAGGGAAGGAGCTA | 105 | 22247 |
| Srebf1c | XM_006532716.2 | GGAGCCATGGATTGCACATTT | CAGCATAGGGGGCGTCAAA | 91 | 3078 |
| Tbp | NM_013684.3 | AGCCTTCCACCTTATGCTCAG | GCCGTAAGGCATCATTGGACT | 90 | 1145 |
